# Supplementary material for: Utility of machine learning in developing a predictive model for early-age-onset colorectal neoplasia using electronic health records
Source: PLoS One. 2022 Mar 10;17(3):e0265209. doi: 10.1371/journal.pone.0265209 (PMC9064446; doi:10.1371/journal.pone.0265209)
Supplement: S1 Table — (DOCX) [file pone.0265209.s001.docx]

**S1 Table: Comparison of predictors in a sub-sample of patients aged 46–49 undergoing diagnostic and screening colonoscopy**

|  | Screening indication | Diagnostic indications | *P-value* | Missing data |
| --- | --- | --- | --- | --- |
| Total number of included patients | 296 | 407 |  |  |
| Birth year [mean (S.D.)] | 1970.3 (1.4) | 1970.4 (1.2) | 0.071 | 0.0 |
| Age [mean (S.D.)] | 48.3 (1.2) | 48.0 (1.1) | < 0.001 | 0.0 |
| Female gender | 58.4% | 59.0% | 0.951 | 0.0 |
| Race |  |  | 0.567 | 3.1 |
| Hispanic | 2.4% | 2.9% |  |  |
| Asian | 1.7% | 2.0% |  |  |
| African American | 24.7% | 20.6% |  |  |
| Non-Hispanic White | 62.2% | 61.4% |  |  |
| Other | 6.8% | 9.3% |  |  |
| NA | 2.4% | 3.7% |  |  |
| Rural-urban commuting area code (RUCA2) |  |  |  |  |
| Mean (S.D.) | 1.3 (1.28) | 1.3 (1.14) | 0.630 | 0.0 |
| Metropolitan [RUCA 1 – 3] | 92.9% | 93.6% | 0.999 | 0.0 |
| Micropolitan [RUCA 4 – 6] | 4.7% | 4.7% |  |  |
| Small town [RUCA 7 – 9] | 2.4% | 1.7% |  |  |
| Rural [RUCA 10] | 0.0% | 0.0% |  |  |
| Returns within income brackets per zip code [mean (S.D.)] |  |  |  | 0.3 |
| $1 to under $25,000 | 30.4% (9.3) | 31.0% (9.7) | 0.403 |  |
| $25,000 to under $50,000 | 24.6% (7.3) | 24.7% (6.9) | 0.785 |  |
| $50,000 to under $75,000 | 14.5% (2.6) | 14.6% (2.6) | 0.589 |  |
| $75,000 to under $100,000 | 9.4% (2.8) | 9.5% (3.0) | 0.473 |  |
| $100,000 to under $200,000 | 14.8% (7.9) | 14.4% (7.7) | 0.480 |  |
| $200,000 or more | 6.1% (6.0) | 5.5% (5.6) | 0.166 |  |
| $1 to under $100,000 | 79.0% (13.3) | 80.0% (12.6) | 0.294 |  |
| $100,00 to under $200,000 | 20.9% (13.3) | 19.9% (12.6) | 0.294 |  |
| $1 to under $50,000 | 55.0% (15.7) | 55.8% (15.5) | 0.523 |  |
| $50,000 to under $100,000 | 23.9% (4.8) | 24.2% (5.2) | 0.480 |  |
| ≥ $100,000 | 20.9% (13.3) | 19.9% (12.6) | 0.294 |  |
| Single tax returns per zip code | 48.4% (7.93) | 49.18% (9.21) | 0.285 | 0.3 |
| Adjusted gross income per zip code | $1,395,095.35 ($862,390.1) | $1,322,105.92 ($849,466.7) | 0.265 | 0.3 |
| ASA categories |  |  |  |  |
| ASA I (healthy patient) | 28.0% | 17.0% | 0.98 | 0.0 |
| ASA II (mild systemic disease) | 65.9% | 73.0% |  |  |
| ASA III (severe systemic disease) | 6.1% | 10.1% |  |  |
| ASA IV (life threatening systemic disease) | 0.0% | 0.0% |  |  |
| ASA ≤ II vs > II |  |  | 0.081 | 0.0 |
| Symptoms/Indication |  |  |  |  |
| Weight loss |  |  | 0.017 | 0.0 |
| Yes | 0 | 2.5% |  |  |
| No | 100% | 97.5% |  |  |
| Abdominal pain |  |  | < 0.001 | 0.0 |
| Yes | 0 | 21.6% |  |  |
| No | 100% | 78.4% |  |  |
| Gastrointestinal bleeding |  |  | < 0.001 | 0.0 |
| Yes | 0% | 40.5% |  |  |
| No | 100% | 59.5% |  |  |
| Anemia |  |  | < 0.001 | 0.0 |
| Yes | 0 | 10.1% |  |  |
| No | 100% | 89.9% |  |  |
| Diarrhea (%) |  |  | < 0.001 | 0.0 |
| Yes | 0% | 5.7% |  |  |
| No | 100% | 94.3% |  |  |
| Constipation |  |  | < 0.001 | 0.0 |
| Yes | 0.7% | 13.8% |  |  |
| No | 99.3% | 86.2% |  |  |
| Change in bowel habits |  |  | < 0.001 | 0.0 |
| Yes | 0% | 6.6% |  |  |
| No | 100% | 93.4% |  |  |
| Rectal pain |  |  | 0.060 | 0.0 |
| Yes | 0% | 1.7% |  |  |
| No | 100% | 98.3% |  |  |
| Pelvic pain |  |  | 0.371 | 0.0 |
| Yes | 0% | 0.7% |  |  |
| No | 100% | 99.3% |  |  |
| Obstipation |  |  | 1.000 | 0.0 |
| Yes | 0% | 0.2% |  |  |
| No | 100% | 99.8% |  |  |
| Colorectal neoplasm in distant relative |  |  | 0.814 | 0.0 |
| Yes | 3% | 2.5% |  |  |
| No | 97% | 2.5% |  |  |
| Irritable bowel syndrome |  |  | 0.371 | 0.0 |
| Yes | 0% | 0.7% |  |  |
| No | 100% | 99.3% |  |  |
| Change in stool caliber |  |  | 0.060 | 0.0 |
| Yes | 0% | 1.7% |  |  |
| No | 100% | 98.3% |  |  |
| Prior diverticulitis |  |  | 0.001 | 0.0 |
| Yes | 0% | 4.2% |  |  |
| No | 100% | 95.8% |  |  |
| Family history of cancer other than CRC |  |  | 1.000 | 0.0 |
| Yes | 1% | 1% |  |  |
| No | 99% | 99% |  |  |
| Family history of cancer other than CRC |  |  | Not applicable | 0.0 |
| Yes | 0% | 0% |  |  |
| No | 100% | 100% |  |  |
| Height in feet [mean (S.D.)] | 5.6 (0.3) | 5.5 (0.3) | 0.096 | 0.3 |
| Weight in pounds [mean (S.D.)] | 194.5 (52.5) | 190.8 (51.4) | 0.381 | 6.7 |
| BMI (kg/m^2^) |  |  |  | 6.7 |
| Mean (S.D.) | 30.2 (7.5) | 30.06 (7.0) | 0.749 |  |
| ≥ 25 Kg/m^2^ | 65.9% | 75.2% | 0.005 |  |
| < 25 Kg/m^2^ | 24.3% | 20.4% |  |  |
| ≥ 30 Kg/m^2^ | 38.5% | 43.0% | 0.016 |  |
| < 30 Kg/m^2^ | 51.7% | 52.6% |  |  |
| ≥ 35 Kg/m^2^ | 19.3% | 16.2% | 0.007 |  |
| < 35 Kg/m^2^ | 70.9% | 79.4% |  |  |
| ≥ 40 Kg/m^2^ | 9.5% | 8.8% | 0.017 |  |
| < 40 Kg/m^2^ | 80.7% | 86.7% |  |  |
| First quartile [16.5, 25.4] | 25.3% | 21.9% | 0.008 | 6.7 |
| Second quartile [25.4, 29] | 22.0% | 24.3% |  |  |
| Third quartile [29, 33.6] | 18.6% | 26.8% |  |  |
| Forth quartile [33.6, 57.8] | 24.3% | 22.6% |  |  |
| Alcohol use |  |  | 0.252 | 0.7 |
| Never | 1.7% | 1.2% |  |  |
| No | 31.4% | 38.6% |  |  |
| Not currently | 3.0% | 3.2% |  |  |
| Yes | 63.5% | 56.0% |  |  |
| Alcohol use: “Yes” vs. others |  |  | 0.099 | 0.7 |
| Alcohol use: “Never” vs. others |  |  | 0.533 | 0.7 |
| Tobacco use |  |  |  |  |
| Never | 67.9% | 230 (56.5) | 0.017 | 0.3 |
| Passive | 0.0% | 0.2% |  |  |
| Quit | 15.9% | 24.1% |  |  |
| Yes | 16.2% | 18.7% |  |  |
| Tobacco use: “Yes” vs. others |  |  | 0.329 | 0.3 |
| Tobacco use: “Never” vs. others |  |  | 0.006 | 0.3 |
| Intravenous drug user |  |  |  |  |
| No | 99.3% | 98.5% | 0.531 | 1.0 |
| Yes | 0.0% | 0.2% |  |  |
| Illicit drug user |  |  |  |  |
| Never | 7.1% | 3.9% | 0.161 | 1.0 |
| No | 84.1% | 84.5% |  |  |
| Not currently | 3.0% | 2.2% |  |  |
| Yes | 5.1% | 8.1% |  |  |
| Illicit drug user: “Yes” vs. others |  |  | 0.214 | 1.0 |
| Other | 94.3% | 90.7% |  |  |
| Yes | 5.1% | 8.1% |  |  |
| Illicit drug user: “Never” vs. other |  |  | 0.142 | 1.0 |
| Never | 7.1% | 3.9% |  |  |
| Other | 92.2% | 94.8% |  |  |
| Total cholesterol (mg/dL) |  |  |  |  |
| Mean (S.D.) | 182.5 (36.2) | 189.8 (41.9) | 0.047 | 32.4 |
| ≥ 200 mg/dL | 21.3% | 24.8% | 0.097 | 32.4 |
| < 200 mg/dL | 49.0% | 40.8% |  |  |
| ≥ 170 mg/dL | 45.3% | 44.5% | 0.316 | 32.4 |
| < 170 mg/dL | 25.0% | 21.1% |  |  |
| First quartile [88, 160] | 19.9% | 14.7% | 0.258 | 32.4 |
| Second quartile [160,184] | 18.9% | 17.2% |  |  |
| Third quartile [184, 214] | 16.9% | 16.0% |  |  |
| Forth quartile [214, 377] | 14.5% | 17.7% |  |  |
| HDL (mg/dL) |  |  |  |  |
| Mean (S.D.) | 52.5 (16.7) | 52.0 (17.7) | 0.740 | 32.7 |
| ≥ 35 mg/dL | 63.5% | 58.7% | 0.415 | 32.7 |
| < 35 mg/dL | 6.4% | 6.6% |  |  |
| ≥ 40 mg/dL | 55.7% | 50.6% | 0.370 | 32.7 |
| < 40 mg/dL | 14.2% | 14.7% |  |  |
| First quartile [14, 41] | 19.9% | 17.9% | 0.326 | 32.7 |
| Second quartile [41, 49] | 14.2% | 17.0% |  |  |
| Third quartile [49, 60] | 16.9% | 16.2% |  |  |
| Forth quartile [60, 140] | 18.9% | 14.3% |  |  |
| LDL (mg/dL) |  |  |  |  |
| Mean (S.D.) | 105.64 (33.73) | 107.68 (34.15) | 0.519 | 33.4 |
| ≥ 100 mg/dL | 38.2% | 39.6% | 0.251 | 33.4 |
| < 100 mg/dL | 30.7% | 25.3% |  |  |
| ≥ 150 mg/dL | 6.1% | 6.4% | 0.494 | 33.4 |
| < 150 mg/dL | 62.8% | 58.5% |  |  |
| First quartile [15, 83] | 17.6% | 16.5% | 0.78 | 33.4 |
| Second quartile [83, 106] | 18.2% | 16.7% |  |  |
| Third quartile [106, 130] | 16.9% | 14.7% |  |  |
| Forth quartile [130, 206] | 16.2% | 17.0% |  |  |
| Triglyceride (mg/ dL) |  |  |  |  |
| Mean (S.D) | 130.09 (100.30) | 163.28 (207.32) | 0.034 | 32.3 |
| ≥150 mg/dL | 17.6% | 24.3% | 0.009 | 32.3 |
| <150 mg/dL | 52.7% | 169% |  |  |
| First quartile [26, 77] | 20.9% | 14.7% | 0.009 | 32.3 |
| Second quartile [77, 115] | 17.9% | 16.0% |  |  |
| Third quartile [115, 170] | 19.3% | 15.0% |  |  |
| Forth quartile [170, 2870] | 12.2% | 20.1% |  |  |
| Triglyceride: HDL ratio (insulin resistance) |  |  |  |  |
| Mean (S.D.) | 2.97 (2.98) | 3.88 (6.58) | 0.067 | 32.7 |
| High (ratio ≥ 3) | 68 ( 23.0) | 110 ( 27.0) | 0.072 | 32.7 |
| Low (ratio < 3) | 139 ( 47.0) | 156 ( 38.3) |  |  |
| First quartile [0.338, 1.33] | 19.6% | 15.0% | 0.04 | 32.7 |
| Second quartile [1.33, 2.31] | 20.6% | 14.0% |  |  |
| Third quartile [2.31, 3.94] | 15.2% | 17.9% |  |  |
| Forth quartile [3.94, 81.9] | 14.5% | 18.4% |  |  |
| Hemoglobin (mg/dL) |  |  |  |  |
| Mean (S.D.) | 13.75 (1.66) | 13.53 (1.75) | 0.194 | 40.5 |
| First quartile [5.3,12.6] | 12.2% | 18.2% | 0.09 | 40.5 |
| Second quartile [12.6,13.7] | 14.2% | 14.7% |  |  |
| Third quartile [13.7,14.8] | 18.6% | 12.8% |  |  |
| Forth quartile [14.8,17.7] | 13.5% | 14.5% |  |  |
| Non-steroidal anti-inflammatory drugs use |  |  | 0.003 | 0.0 |
| Yes | 8.8% | 17.0% |  |  |
| No | 91.2% | 83.0% |  |  |
| Statin user |  |  | 0.809 | 0.0 |
| Yes | 14.5% | 15.5% |  |  |
| No | 85.5% | 84.5% |  |  |
