# Supplementary material for: Utility of machine learning in developing a predictive model for early-age-onset colorectal neoplasia using electronic health records
Source: PLoS One. 2022 Mar 10;17(3):e0265209. doi: 10.1371/journal.pone.0265209 (PMC9064446; doi:10.1371/journal.pone.0265209)
Supplement: Attachment — Submitted filename: Response to reviewers.docx [file pone.0265209.s002.docx]

Dear Editors at the PLOS ONE Journal:

My coauthors and I would like to thank you for the opportunity to revise our paper. We appreciate the time and effort expended by the reviewers and editorial staff to assist us in the goal of making this paper worthy of publication. We have addressed the concerns of the reviewers (see below and in yellow highlight in the marked version of the manuscript). We also further enhanced the quality of our manuscript and made sure it meets PLOS ONE's style requirements.

Again, we appreciate the opportunity to work with you in the editorial process.

Sincerely yours,

The Authors

**Reviewers’ comments:**

**Comments to the Author**

**Reviewer #1:**

1. **The authors discussing machine learning in this paper but there isn't any clear definition about it. If the authors can provide a clear definition of machine learning and how these machine learning have been use at the introduction section that would helpful**

**Answer:** Thank you for this important point. We included a definition of machine learning with a reference in introduction. A description of the utilized machine learning algorithms is also detailed in the methods and in our discussion.

2. **As the study involved 3,116 adults aged 35-60, I would be interested to know how these sample were recruited for the study**

**Answer:** This is a very good comment. As described, this is a retrospective cohort available at the Ohio State University. We clarified further that our cohort and their colonoscopy data were obtained from the Ohio State University colonoscopy quality reporting GI Quality Improvement Consortium, Ltd (GIQuic) database. GIQuic is a collaborative, nonprofit, scientific organization between the American College of Gastroenterology and the American Society for Gastrointestinal Endoscopy^1^. We linked to electronic health records data to our database to form a retrospective sample of 3,116 adults aged 35-50 with EHR data and colonoscopy-based colorectal neoplasia outcomes. We clarified this point in our methods and Fig 1.

3. **Overall, the paper has demonstrated clear methodology of research**

**Answer:** Thank you for the kind comment.

**Reviewer #2:**

**Dear Authors,
The manuscripts is well-organized and well written. The topic is interesting and significant in the current situation. I would suggest few minor revisions to enhance the quality of your manuscript.**

1. **You have added some supporting information. However, the graphs and charts are not clearly visible. Is it possible to upload better quality image?**

**Answer:** We apologize the quality. It appears the images lost quality in the submission process and we have since rectified this mistake.

2. **The references you used are good enough in quantity, however, it would be beneficial to add few more references which are up to date (preferably from 2020-2022). It will ensure the research gap.**

**Answer:** Thank you for pointing that out. We added more up-to-date references. We also revised and enhanced the quality of the paper.

3. **Please add the managerial and practical significance of the research in a separate paragraph.**

**Answer:** This is a very reasonable comment. We included practical significance of this work in one paragraph.

**Thank you and good luck!**

**Reviewer #3:**

**I am pleased to have an opportunity to review this scientific paper about the predictive models for early-age-onset
colorectal neoplasia. I think this paper is scientifically informative and interesting. I have some comments and suggestions as below.**

1. **Major, the author should add the work plot in this manuscript and it's necessary to add the inclusion and exclusion criteria in this plot.**

**Answer:** Thank you for helping improve the presentation of our paper. We added a plot describing our inclusion and exclusions (Fig 1).

2. **The figure legend and table should put in the end of manuscript.**

**Answer:** Thank you. We are following PLOS ONE's style requirements when it comes to figure legends and tables. Specifically, per the instructions:

“Each figure caption should appear directly after the paragraph in which they are first cited.”

“Tables should be included directly after the paragraph in which they are first cited.”
Link to instructions: [https://journals.plos.org/plosone/s/file?id=wjVg/PLOSOne_formatting_sample_main_body.pdf](https://urldefense.com/v3/__https:/journals.plos.org/plosone/s/file?id=wjVg*PLOSOne_formatting_sample_main_body.pdf__;Lw!!Cjnu1T6GFXg!4DjMDGsQd_3urkcXaIQo_zN7jrNFg9YOWpzpW0KTQv_lfGc9crBIhJfAdCxOio2PgviJ$)

3. **You should put the P value in your figure.**

**Answer:** Many thanks. To clarify, the *p* values compare the machine learning methods to conventional regressions and are already included in Fig 3. We clarified the meaning of our *p* values in Fig 3 in the manuscript. Fig 2 and 4 do not have *p* values to insert.

4. **The history of colorectal neoplasia in family member may be added in this manuscript, which was an important factor associated with colorectal cancer.**

**Answer:** As early-onset CRC primarily occurs in adults with no strong familial predisposition or pre-existing colitis^2^, we included only average-risk adults without family history of CRC. History of colorectal neoplasia was indeed an included factor in our prediction module, but however it was not high enough compared to environmental risk factors such as social factors, obesity and insulin resistance. This is likely since environmental risk factors are likely more contributing than genetic factors for the increased risk in average-risk adults with early-onset neoplasia.

5. **Patients with abnormal laboratory studies (CEA and CA199) should be excluded in this manuscript which may result a bias of the cohort.**

**Answer:** We completely agree. None of the patients in the cohort were referred due to an abnormal CEA or CA199. We clarified that in our exclusions.

**REFERENCES:**

1.	GIQuIC. [www.giquic.org](http://www.giquic.org). Accessed January 27, 2022.

2.	Dozois EJ, Boardman LA, Suwanthanma W, et al. Young-onset colorectal cancer in patients with no known genetic predisposition: can we increase early recognition and improve outcome? *Medicine (Baltimore).* 2008;87(5):259-263.
